# Construction of a ternary component chip with enhanced desorption efficiency for laser desorption/ionization mass spectrometry based metabolic fingerprinting

**DOI:** 10.3389/fbioe.2023.1118911

**Published:** 2023-01-20

**Authors:** Yajie Ding, Congcong Pei, Kai Li, Weikang Shu, Wenli Hu, Rongxin Li, Yu Zeng, Jingjing Wan

**Affiliations:** ^1^ School of Chemistry and Molecular Engineering, East China Normal University, Shanghai, China; ^2^ Department of Urology, Tianjin Third Central Hospital Affiliated to Nankai University, Tianjin, China

**Keywords:** mass spectrometry, on-chip analysis, mesoporous silica membrane, metabolomics, kidney stone

## Abstract

**Introduction:** *In vitro* metabolic fingerprinting encodes diverse diseases for clinical practice, while tedious sample pretreatment in bio-samples has largely hindered its universal application. Designed materials are highly demanded to construct diagnostic tools for high-throughput metabolic information extraction.

**Results:** Herein, a ternary component chip composed of mesoporous silica substrate, plasmonic matrix, and perfluoroalkyl initiator is constructed for direct metabolic fingerprinting of biofluids by laser desorption/ionization mass spectrometry.

**Method:** The performance of the designed chip is optimized in terms of silica pore size, gold sputtering time, and initiator loading parameter. The optimized chip can be coupled with microarrays to realize fast, high-throughput (∼second/sample), and microscaled (∼1 μL) sample analysis in human urine without any enrichment or purification. On-chip urine fingerprints further allow for differentiation between kidney stone patients and healthy controls.

**Discussion:** Given the fast, high throughput, and easy operation, our approach brings a new dimension to designing nano-material-based chips for high-performance metabolic analysis and large-scale diagnostic use.

## 1 Introduction

*In vitro* diagnostics (IVD) contributes to about 70% of clinical diagnoses, thereby often referring to the “eyes” of doctors (Rohr et al., 2016; Wishart, 2016). For IVD, metabolic profiling is more distal over proteomic and genomic analysis (Häkkinen, 2012; Lee et al., 2018), so it has been widely applied in biomedical research and clinical practice (Nicholson et al., 2012; Aboud and Weiss, 2013; Nemet et al., 2020). Mass spectrometry (MS) with high throughput, sensitivity, and accuracy has been recognized as a new tool for metabolite analysis (Griffiths et al., 2010; Stolee et al., 2012; Zenobi, 2013; Wang et al., 2022). Among the ever-developing MS technologies, laser desorption/ionization mass spectrometry (LDI MS) has attracted intense attention in the field of metabolic diagnosis, considering its microliter sample requirements and second-level detection speed (Huang et al., 2017; Li et al., 2021; Ding et al., 2022; Shu et al., 2022). Metabolites are mixed with UV-absorbing materials called a matrix, and the UV laser irradiation of the mixtures promotes the efficient desorption and soft ionization of the metabolites (Wu et al., 2016; Wu et al., 2017; Pei et al., 2020; Pei and Wan, 2020; Wang et al., 2020; Yang et al., 2020). However, the enhancement of desorption/ionization efficiency has been a major bottleneck for its routine application in clinical practices (Lin et al., 2015; Liu et al., 2016; Cao et al., 2020). Another bottleneck for LDI MS in diagnostic is the poor reproducibility raised from the un-uniform matrix and sample distribution *via* manual workflows (Hu et al., 2013; Joh et al., 2021). The chip design can minimize the sample pre-treatment process in LDI detection *via* matrix pre-integration and laboratory automation (Sun et al., 2018; Shu et al., 2020). Hence, the design of LDI MS chip is promising to address these challenges if the following aspects can be realized: 1) precisely designed matrix formula for selective and sensitive detection of metabolites in complex biological fluids; 2) functional interface improving desorption/ionization efficiency and background noises; 3) combination with microarray technology and machine learning for real case applications. Currently, more researchers focus on the matrix in LDI MS-based metabolic diagnosis, but only a few studies of LDI MS chips have been reported.

The major components of an LDI-MS chip are substrates as the skeleton, matrices for efficient laser energy transfer, and surface modifiers for functionalization (Lim et al., 2012; Sun et al., 2018; Shu et al., 2020). Among them, matrices are considered the most fundamental parameters in metabolic analysis, in which inorganic materials with stable structures and UV-absorbing are preferred organic compounds with strong background interference (Noh et al., 2017; Kim et al., 2021). Existing matrices are mostly based on plasmonic metal (Du et al., 2019; Wang et al., 2021; Yin et al., 2022), metal oxides (Kim et al., 2020), carbon (Coffinier et al., 2012), and silicon (Gao et al., 2016; Korte et al., 2016). Typically, metals (e.g., Au) with pre-selected structures display surface plasmon resonance and generate hot carriers under laser irradiation, serving as an emerging class of matrix materials in chip design (Sun et al., 2018; Wang et al., 2020). Our group recently demonstrated a dopamine bubble-based approach for efficient plasmonic gold growth on an indium tin oxide (ITO) chip (Shu et al., 2020). Besides the matrices, the modifier, also called initiator, plays an essential role in LDI detection, as it can enhance the non-thermally driven desorption and reduce background interference (Northen et al., 2007; Kurczy et al., 2015; Gao et al., 2016). Furthermore, the introduced hydrophobic surface also effectively suppressed the excessive spread of a droplet of aqueous sample solution on the solid substrate (Palermo et al., 2018). By integrating the most common perfluoroalkyl initiator with plasmonic metal, a highly efficient matrix formula has been achieved for sensitive metabolite imaging on brain tissues. Despite that, the developed protocol for initiators loading on metal is highly hazardous (using HF) or cumbersome and non-compatible with feasible MS chip production for large-scale use in IVD.

Unlike the extensively studied matrix formula, the chip substrate is less investigated but valued. The substrate can either be the skeleton for matrices and modifiers or be employed as the matrix (Northen et al., 2007; Nakamura and Soejima, 2019; Jiang et al., 2021). Of note, the nanostructure of the substrate has an impact on the desorption/ionization process in LDI detection (Ng et al., 2015). In a plausible LDI mechanism, it is crucial to reserve the heat energy generated by laser irradiation, as the thermal energy assists the desorption and evaporation of the analytes (Dreisewerd, 2003). Mesoporous structures with low thermal conductivity (Yan et al., 2019) can achieve a high extent of internal-energy transfer from the chip to the analytes, resulting in enhanced thermally driven desorption. Therefore, the mesoporous structure displays excellent potential in the chip substrate design and is realized in the silicon and aluminum oxide arrays (Nayak and Knapp, 2007; Xiao et al., 2009). In contrast to those conventional substrates, silica serves as an ideal candidate for LDI chip substrate due to the low thermal conductivity (7.6 W m/(m·k)), high chemical stability, low background interference, and adjustable pore structure (Zhao et al., 2022). Very recently, Norihiro et al. reported nanoporous organosilica membranes with a pore size of ∼150 nm as a novel substrate for small protein detection in LDI-MS (Mizoshita et al., 2022). While to achieve a direct and sensitive detection of small metabolites (<5 nm) in bio-fluids, the mesoporous silica substrate needs to be carefully designed to couple with the LDI-MS chip for high sensitivity and selectivity.

**SCHEME 1:**
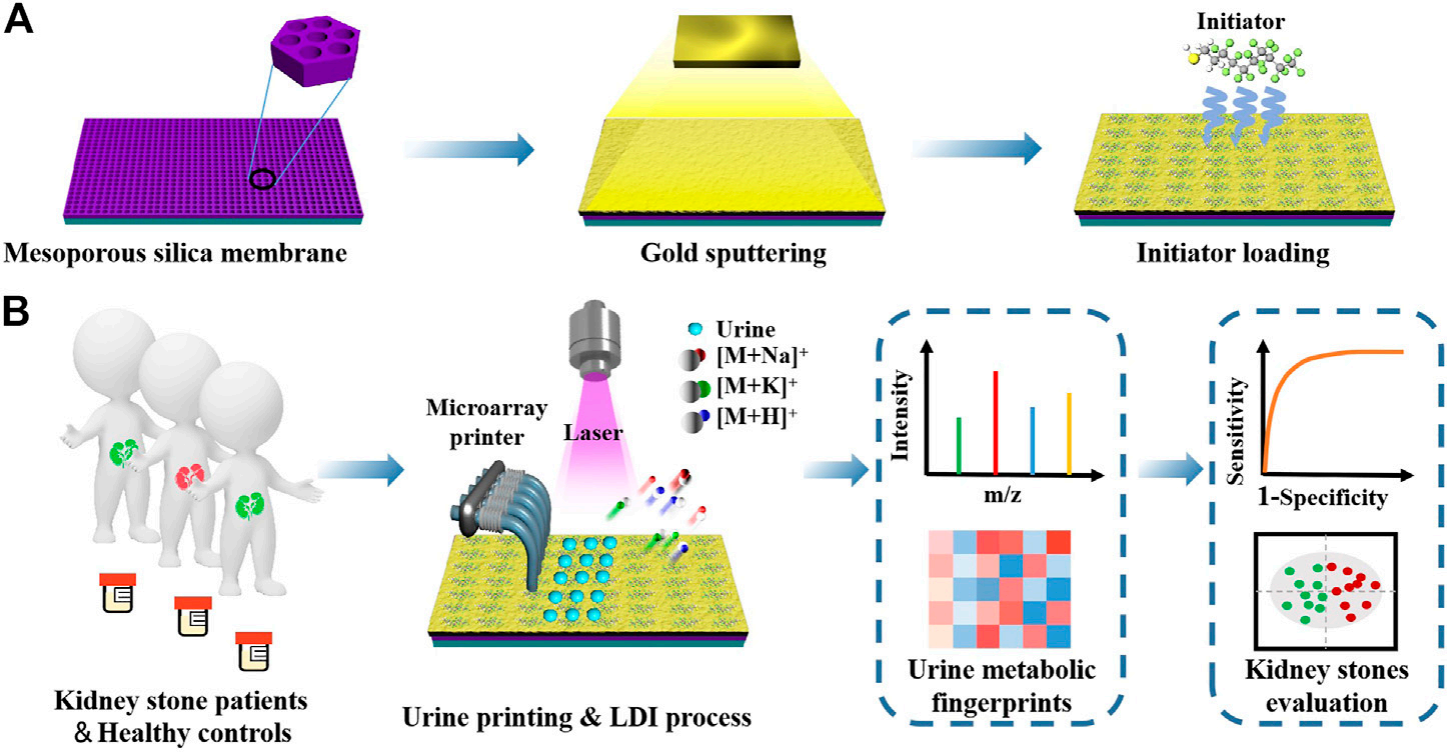
Schematic illustrations of **(A)** synthetic route of initiator/gold/mesoporous silica membrane chips (IGMSM chips) and **(B)** LDI-MS extraction of urine metabolic fingerprints by IGMSM chips for evaluation of kidney stone.

In this work, we developed a ternary component MS chip composed of mesoporous silica substrate, plasmonic gold matrix, and perfluoroalkyl initiator for direct metabolic fingerprinting of biofluids in diagnostics (Scheme 1). It is well known that the LDI efficiency is highly determined by the thermally and non-thermally driven desorption. By tuning the silica pore size, gold sputtering time, and initiator incorporating method, the constructed chip brought in an increased internal energy transfer for the enhanced thermal desorption and generated more violent phase-transition of the gold and initiator for enhanced non-thermal desorption, thereby enabling fast, sensitive, and selective detection of small metabolites in human urine without any pre-enrichment or pre-purification. The optimized chip can be coupled with microarrays to realize high-throughput (∼second/sample) and microscaled (∼1 μL) sample analysis. For disease detection, we demonstrated on-chip *in vitro* metabolic diagnosis of kidney stones patients using urine with an area under the curve (AUC) value of 0.912. This work contributes to designing nano-material-based platforms for high-performance metabolic analysis and large-scale diagnostic use.

## 2 Materials and methods

### 2.1 Reagents

Cetyltrimethylammonium chloride (CTAC), triethanolamine (TEA), 1H,1H,2H, 2H-perfluorodecanethiol, pyridine, benzyl chloride, and cyclohexane were purchased from Sigma-Aldrich Corp. Sodium chloride, sodium hydroxide, tetraethyl orthosilicate (TEOS), methanol ethanol, bovine serum albumin (BSA) proline, mannitol, lysine, and sucrose were purchased from Adamas Reagent Ltd. All the above chemicals were used as received without further purification. ITO slides were purchased from Luoyang Guluo Glass Co., Ltd. These ITO slides were first treated with NaOH solution (2 M) at room temperature for 12 h to clean the organic residues and then stored in deionized water for the subsequent experiments. Gold sputtering material (99.99%) was purchased from Hefei Kejing Auto-instrument Co., Ltd, China. Deionized water (18.2 MΩ cm) was obtained by a Milli-Q system (Millipore, United States) and used for all experiments in this work.

### 2.2 Chip synthesis

#### 2.2.1 Synthesis of the chips with mesoporous silica membranes

According to the reported literature (Liu et al., 2017), 20 wt% CTAC solution was prepared in advance and used after standing for more than 12 h 30 mL of the CTAC solution and 0.36 mL of TEA were added to 70 mL of deionized water. The mixture first was transferred to a 250 mL three-necked flask with an ITO slide on the bottom and stirred gently at 60°C for 1 h. Then, 34 mL of TEOS in cyclohexane (5 v/v %) was dropwise added into the above mixture at 60°C. The reaction was continued at a constant temperature with continuous stirring for another 2.5 h. Later, the chip was taken out and washed with methanol, ethanol, and deionized water, and then dried at 60°C for 1 h. After removing the surfactant templates by simple calcination at 500°C for 3 h in air, chips with mesoporous silica membranes were obtained.

#### 2.2.2 Gold sputtering

The sputtering was conducted by a VTC-1RF magnetic control sputtering coater (Hefei Kejing Auto-instrument Co., Ltd, China) at the current of 7.8 mA under vacuum for 30–60 s. With different sputtering times (30–60 s), the content of gold and surface structure of chips can be tuned. The sputtering angle was kept to be vertical in all cases and the system pressure for sputtering was 18 Pa.

#### 2.2.3 Initiator loading

Put the chip with the gold layer in a glass Petri dish with a lid. Then added 5 μL of 1H,1H,2H,2H-perfluorodecanethiol to the Petri dish and covered it with the lid. After putting the Petri dish in an oven at 80°C for 30–90 s, the chips modified with different amounts of initiators were obtained.

### 2.3 Synthesis of thermometer molecules and related definitions

Ion desorption efficiency was gauged by the chemical thermometer (benzyl pyridinium (BP)). 100 μL of Benzyl chloride was mixed with 2 mL pyridine and stirred in a 50 mL round-bottomed flask at 60°C for 5 h. Then the excess pyridine was removed by rotary evaporation. The end-product was redissolved in 1:1 (v/v) MeOH: H_2_O to prepare a 0.1 mM [BP]^+^ solution.

When the internal energy of [BP]^+^ (parent ions) is larger than the dissociation reaction critical energy, the parent ions would undergo bond cleavage:

$${\left[BP\right]}^{+}\;\to \;{\left[\;BP-pyridine\;\right]}^{+}+pyridine$$

m/z170 m/z91 

The total intensity of BP ions includes the intensity of parent ions and [ BP—pyridine]^+^ (fragment ions). A higher total ion intensity indicates a higher desorption efficiency. The survival yield (SY) of parent ions is used to gauge the degree of parent ions fragmentation from internal-energy transfer.

$$SY=\left[I_{m/z\;170}\;/\;\left(I_{m/z170}+I_{m/z91}\right)\right]\times 100\%$$

### 2.4 Characterization

Scanning electron microscopy (SEM) images of chips were obtained from an S4800 field emission scanning electron microscope (Hitachi, Japan). Elemental mapping and energy-dispersive X-ray spectroscopy (EDX)of chips were obtained from a GeminiSEM450 field emission scanning electron microscope (Zeiss, Germany). Transmission electron microscopy (TEM) and selected area electron diffraction (SAED) were obtained from a JEM-2100F transmission electron microscope (JEOL, Japan). Digital images were captured by iQOO three plus (VIVO, China). Microscope images were captured by an FV3000 confocal laser scanning microscope (Olympus, Japan). The contact angle was detected by DSAeco device (KRUSS GmbH, Germany) using 5 μL of water. Inductively coupled plasma optical emission spectrometer (ICP-OES) data was collected by Agilent 5,100 (Agilent Technologies Inc, United States).

### 2.5 Bio-samples harvesting, microarray printing and on-chip LDI MS analysis

All of the research protocols in this study were approved by the institutional ethics committees of Tianjin Third Central Hospital Affiliated with Nankai University and East China Normal University, School of Chemistry and Molecular Engineering. Written informed consent from patients had been obtained since the project started. Urines samples were donated by patients from Tianjin Third Central Hospital Affiliated with Nankai University and stored in tubes at −80°C before use. All kidney stone (KS) patients recruited were eligible and precisely diagnosed based on ultrasound and computed tomography. The urine samples of healthy control (HC) were collected from individuals who were not diagnosed with KS through health examinations. Patients with other medical conditions (such as active bleeding) were excluded. To distribute the bio-fluids on the surface of the prepared chips, GeSim Nano-Plotter TM 2.1 was applied. Each sample spot volume was 1 µL by running the designed printing procedure in the non-contract model. After drying at room temperature, bio-sample on the as-printed chips was detected by LDI MS without ant treatment. 1 µL of standard analytes and prepared mixtures were dissolved in deionized water and spotted on the prepared chips. 1 µL of urine samples were directly spotted on the prepared chips.

## 3 Results and discussion

### 3.1 Construction and characterization of the ternary component chip

**FIGURE 1:**
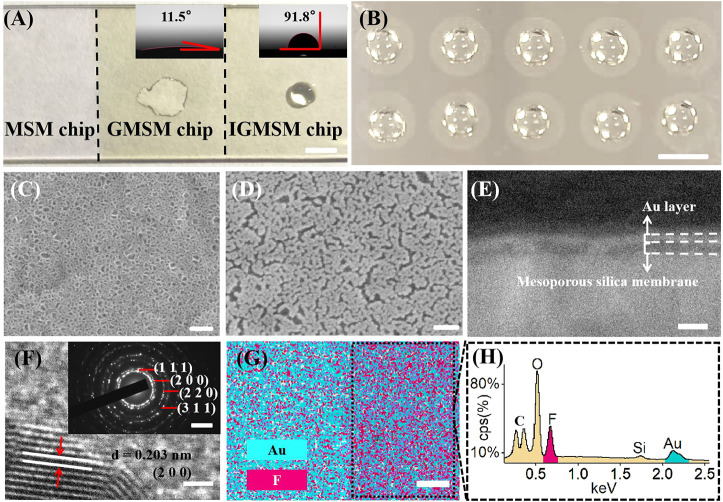
Construction of IGMSM chips: **(A)** Digital image and contact angle (inset) of IGMSM chips (scale bar is 5 mm). **(B)** Digital image of microarray printed chip (scale bar is 1 cm). Top-view image of SEM of **(C)** mesoporous silica membranes on ITO slide and **(D)** gold islands and mesoporous silica membranes on ITO slide (scale bar is 100 nm). **(E)** Cross-section image of SEM of IGMSM chip (scale bar is 100 nm). **(F)** HRTEM (scale bar is 1 nm) showing the gold crystal lattice and SAED pattern (inset) of gold islands (scale bar is 5 1/nm). **(G)** Corresponding element mapping images of the IGMSM chip (scale bar is 100 μm). **(H)** EDS spectra of the area selected in the Figure 1G.

The initiator/gold/mesoporous silica membrane chips (IGMSM chips, meanwhile, denoted mesoporous silica membrane chips as MSM chips and gold/mesoporous silica membrane chips as GMSM chips.) were fabricated through a three-step process on ITO slides including mesoporous silica membranes synthesis, gold sputtering, and initiator loading, facile for ion signal production in LDI-MS (Scheme 1A). The prepared chips with optimized parameters have a uniform and suitable hydrophobicity structure to achieve high reproducibility in practical use (Figure 1A). Meanwhile, the chip is compatible with microarray printing, enabling high-throughput metabolic analysis with bio-samples (Figure 1B). The designed chip can simplify the sample pretreatment process and achieve laboratory automation to realize ideal clinical use. Specifically, the mesoporous silica membranes were evenly distributed on the ITO slides using a biphase stratification growth method reported in the previous research (Liu et al., 2017) to provide substrate for supporting and heat insulation. From the top-view SEM (Figure 1C), the uniform large-domain mesoporous silica membrane has an average pore size of ∼11 nm. Then the gold nanoparticles (AuNPs) were deposited on the silica layer by sputtering as the matrix for LDI-MS. Compared to the MSM chip, gold islands were clearly observed on the top of the GMSM chip from the top-view image of SEM, indicative of the successful sputtering of Au nanoparticles (Figure 1D). The cross-section image of SEM (Figure 1E; Supplementary Figure S1) also showed the silica layer and gold layer on the surface of the ITO slide with a thickness of ∼17 nm and ∼11 nm, respectively. The HRTEM image showed clear fringes with an interplanar distance of 0.203 nm (Figure 1F), which can be ascribed to the (200) plane of Au crystal (JCPDS 00–004–0,784). The SAED pattern gave diffraction spots that correspond to the (111), (200), (220), and (311) lattice planes from Au crystal (Figure 1F). It is well known that AuNPs can be functionalized with a variety of ligands by performing gold - thiol reactions with straightforward synthetic protocols (Häkkinen, 2012; Nyamekye et al., 2021; Zhang et al., 2021). For IGMSM chips, we finally functionalized the surface of AuNPs with 1H,1H,2H, 2H-perfluorodecanethiol chains *via* gold-sulfur coupling, leading to the formation of a highly ordered perfluorinated nanostructured monolayer. No obvious difference can be seen from the SEM images of the chip before and after initiator loading (Figure 1D; Supplementary Figure S2). The contact angle increased from 11.5 to 91.8 through the distribution of the initiators on the gold surface (Figure 1A). The as-made initiators not only improve the non-thermally desorption but also adjust the hydrophobicity of the chip surface for sample droplets spread. From the overlaid element mapping image of the chip, we can observe a clear distribution boundary between the region of non-initiator and initiator (Figure 1G). The EDX spectra of the initiator and non-initiator region further confirm the presence of fluorine from the initiator (Figure 1H; Supplementary Figure S3; Supplementary Figure S4).

### 3.2 Control and optimization of IGMSM chip structural parameters

**FIGURE 2:**
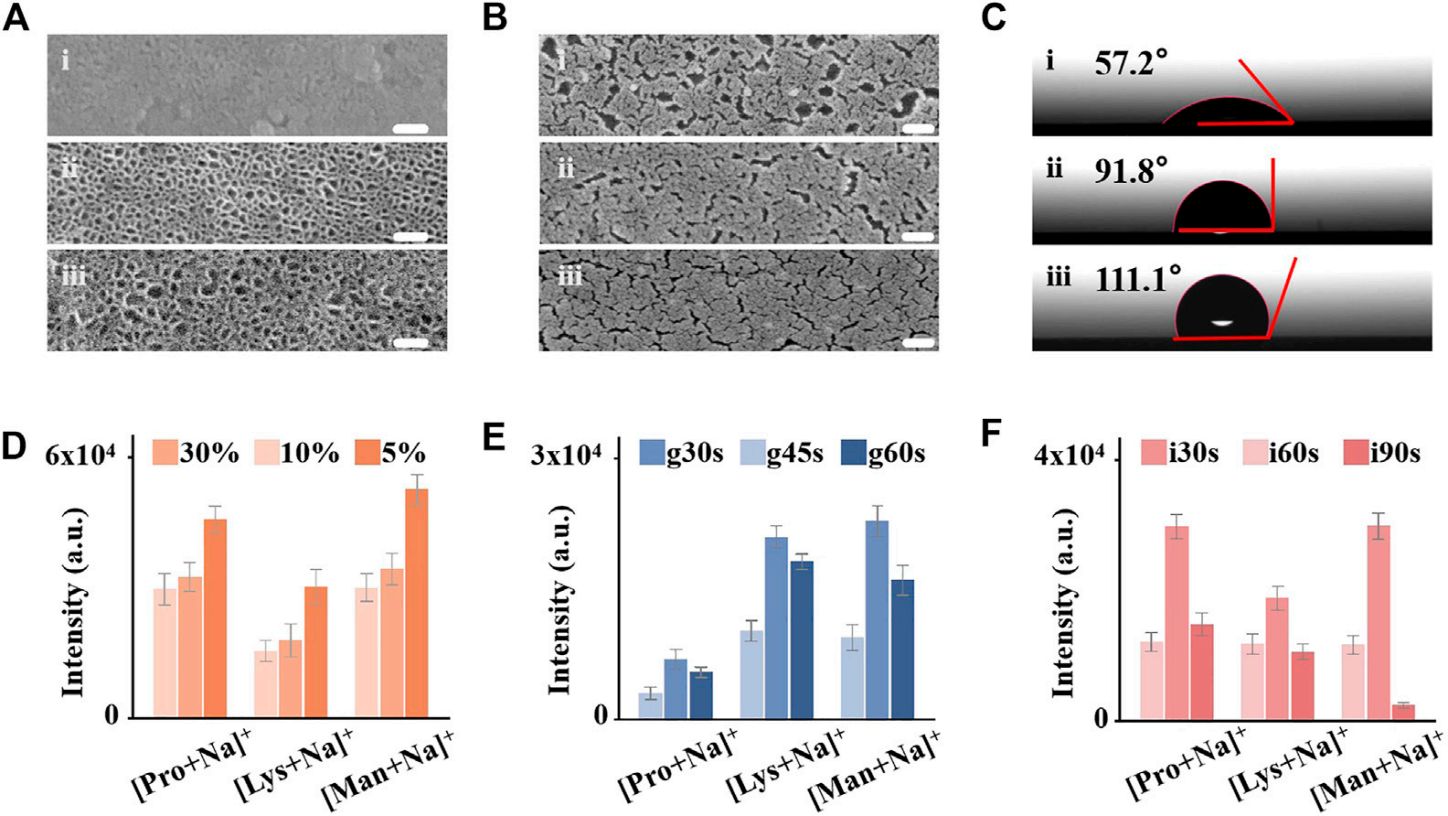
Control and optimization of IGMSM chip structural parameters. **(A)** Top-view image of SEM of mesoporous silica membranes on chip_30%_ 1), chip_10%_ 2), and chip_5%_ 3) (Scale bar is 60 nm). **(B)** Top-view image of SEM of Au islands with different sputtering times on chip_g30s_ 1), chip_g45s_ 2), and chip_g60s_ 3) (Scale bar is 100 nm). **(C)** Contact angles of the chip_i30s_ 1), chip_i60s_ 2), and chip_i90s_ 3). Mean intensities of Na adducted peaks for 1 mg/mL proline, lysine, and mannitol on **(D)** chip_5%/10%/30%_, **(E)** chip_g30s/g45s/g60s_ **(F)** chip_i30s/i60s/i90s_. The error bars were calculated as S.D. of five measurements.

We controlled the conditions of mesoporous silica membrane synthesis, gold sputtering, and initiator loading to optimize the structure parameters of IGMSM chips for excellent LDI performance. For the silica membrane, two parameters are possible to be adjusted including membrane thickness and pore size. Of note, the influence of membrane thickness on thermal conductivity has been investigated in the literature, suggesting that thermal conductivity is independent of thickness. (Coquil et al., 2009). On contrast, mesoporous materials with high porosity exhibit excellent thermal insulation for the desorption process in LDI MS (Nayak and Knapp, 2007; Xiao et al., 2009), and it has been confirmed that the porosity of the mesoporous silica membrane in this study is positively correlated with pore size (Yan et al., 2019). Hence, we tuned the pore size of the mesoporous silica membranes by varying the ratio of TEOS and cyclohexane (v/v %) to get the optimal porosity for thermal insulation. Briefly, the pore size is inversely correlated with TEOS amount. With a smaller content of the TEOS (5%), the mesoporous silica membranes have an average pore size of ∼11 nm (Figure 2A; Supplementary Figure S5), larger than the average pore size of ∼8 nm for 10% (v/v) (Figure 2A). While further increasing the content of TEOS to 30% (v/v), the resultant pore diameters decrease to hardly seen on the chip (Figure 2A). We opted to further decrease the TEOS content below 5%, while the pore became very un-uniform (Supplementary Figure S6). To investigate the impact of the pore size on LDI efficiency, we loaded the three silica chip substrates (denoted chip_5%/10%/30%_) and bare ITO slide with the same amount of gold and initiator for comparison (Supplementary Figure S7; Supplementary Table S1). Then, we conducted LDI-MS detection of three metabolites (proline, lysine, and mannitol) (triplicate results in Figure 2D and typical spectra in Supplementary Figure S8), and chip_5%_ afforded the highest peak intensity and signal-to-noise ratio (*p* < 0.05), indicating that the pore size is positively correlated with the LDI efficiency. Furthermore, the peaks of three amino acids on bare ITO slide were hardly observed (Supplementary Figure S8), suggesting that the mesoporous silica membrane modification enhanced the LDI efficiency, possibly due to better laser energy transfer through the excellent thermal insulation. The detailed thermal-driven desorption process on the mesoporous silica membrane will be discussed later.

The area density of gold on-chip decides the average interparticle distance for plasmon coupling, which is involved in the process of hot carrier production, local heating, and photodesorption for plasmonics enhanced LDI MS. Therefore, we optimized the gold density on the optimal mesoporous silica chip by changing the sputtering time of 30s/45s/60s (denoted chip_g30s/g45s/g60s_). Through the SEM images of the chips with different sputtering times, we observed the increased content of gold and more narrow gaps with longer sputtering time (Figure 2B). We recorded corresponding ICP-OES of the three plasmonic chips (Supplementary Figure S9), in which the gold content increased with the increase of the sputtering time (5.62 μg/cm^2^ for chip_g30s_, 6.32 μg/cm^2^ for chip_g45s_, and 6.65 μg/cm^2^ for chip_g60s_, respectively). From the LDI-MS detection of proline, lysine, and mannitol, we demonstrated that the optimized chip_g45s_ with a gold area density of ∼6.32 μg/cm^2^ afforded specific nanogaps and surface plasmon resonance for highly efficient analysis (triplicate results in Figure 2E and typical spectra in Supplementary Figure S10). The weak ionization efficiency in chip_g30s_ was mainly attributed to the insufficient Au content on the surface. While overloading of gold on the silica membranes (chip_g60s_) also affected their LDI performance, possibly due to the undesired plasmon coupling arising from the overaggregation of gold nanoparticles (Sun et al., 2018; Shu et al., 2020). Furthermore, the specific nanogaps and nanocrevices of gold on-chip_g45s_ may selectively trap small metabolite molecules and transfer the laser energy, toward advanced metabolic analysis of complex biosamples in real cases.

Controlling the surface wettability of the chip is essential for the microscale sample analysis, preventing the lateral spreading of the droplet and localizing the analytes with the desired enrichment. Wettability-controlled IGMSM chips were constructed by adjusting the loading time of the highly hydrophobic initiator (1H,1H,2H, 2H-perfluorodecanethiol) on the optimal gold/mesoporous silica chip, from 0s, 30s, 60s–90s (denoted as chip_i0s/i30s/i60s/i90s_). This perfluoro-initiator trapped in the gold islands can be released when heated by the laser, promoting the generation of intact molecular ions while producing lower background signals (Northen et al., 2007; Kurczy et al., 2015; Palermo et al., 2018). In general, the hydrophobicity increased with the increasing of the initiator amount on the chip. The contact angles of the chip_i0s/i30s/i60s/i90s_ were measured to be 11.5, 57.2, 91.8, and 111.1°, respectively (Figure 2C; Supplementary Figure S11). We used these chips to conduct LDI-MS detection of proline, lysine, and mannitol (triplicate results in Figure 2F, typical spectra in Supplementary Figure S12), and the chip_i60s_ with the contact angle of 91.8 provided the highest peak intensity than the others. Insufficient loading of the initiator remained a hydrophilic surface, leading to the excessive spread of a droplet of aqueous sample solution on the chip and poor reproducibility (Supplementary Figure S13). Overloading the initiator on the IGMSM chips also reduced the analytical efficiency, owing to strong hydrophobicity causing thick samples layer to prevent initiator-assisted desorption of analytes (Woo et al., 2008) (Supplementary Figure S14) and the contact between analytes and gold matrix. Through the three-step optimization, the limit of detection (LOD) of the optimized IGMSM chips in detecting metabolites was down to ∼2 pmol (Supplementary Figure S15) and the relative standard deviations was down to 4.3% (4.4% for proline, 8.7% for lysine and 4.3% for mannitol) when coupled with the routine LDI TOF MS. Hence, compared with previously reported matices and chips, the optimized IGMSM chips with low thermal conductivity, surface plasmon resonance effect, and well wettability enabled the metabolic detection of analytes with desired sensitivity and reproducibility (Supplementary Table S1). Meanwhile, the background signal of the bare IGMSM chip was very low, so it would not interfere with the detection of the analytes (Supplementary Figure S16).

### 3.3 IGMSM chip with enhanced desorption efficiency for real case sample detection

**FIGURE 3:**
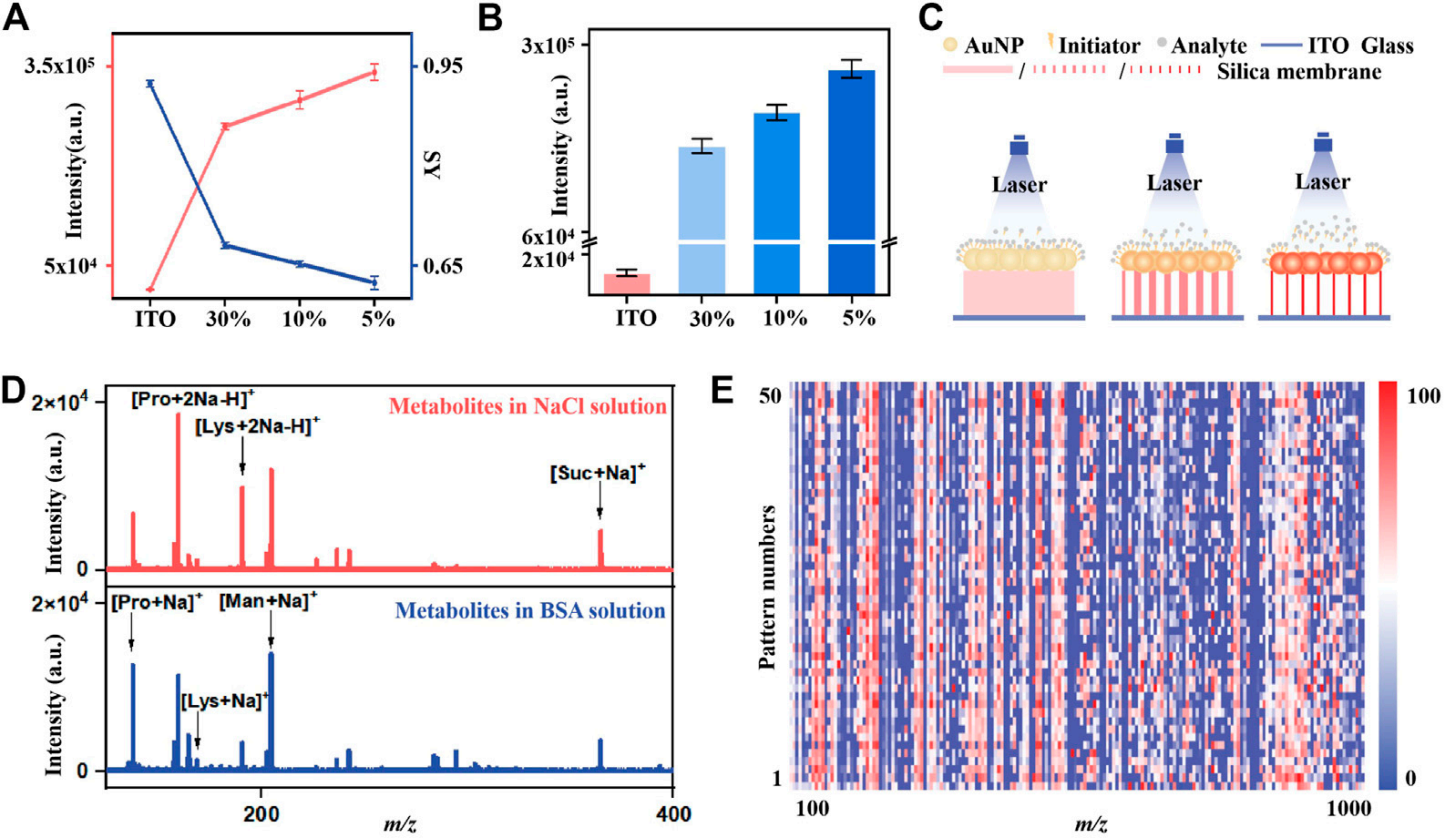
The determination of ion-desorption efficiency and internal energy transfer in the LDI process of chips with different porosity silica membranes: **(A)** The total intensity of BP ions (red line) and SY of parent ions (blue line) desorbed from chips with different porosity silica membranes. **(B)** The intensity of parent BP ions desorbed from chips with different porosity silica membranes **(C)** Schematic illustration of the effect of silica membranes with different porosity on LDI performance. **(D)** The mass spectra of small metabolites mixture in 0.5 M NaCl solution (red) and in 5 mg/mL BSA solution (blue) on IGMSM chip **(E)** Heat map of 50 independent metabolic patterns for one KS patient urine sample within *m/z* range from 100 to 1,000 through data preprocessing.

To better interpret the enhanced LDI performance, we investigated the detailed desorption mechanisms of the IGMSM chip, using BP salts as the “chemical thermometer” (Tang et al., 2009). The mechanistic studies of gold and initiator have been reported by many researchers (Palermo et al., 2018), while the effect of different mesoporous silica membrane substrates on the desorption efficiency remains to be elucidated. With this regard, we chose the IGMSM chips with three different mesoporous silica membranes (chip_5%/10%/30%_) to reveal the possible desorption mechanisms in our design. The detailed definition, calculation of desorption efficiency, and SY are provided in the part 2.3. We plotted desorption efficiency and SY of chips against different porosity of silica membranes. Briefly, the parent ion [BP]^+^ can be dissociated to fragment ion [BP - pyridine]^+^ in the LDI process, and the desorbed BP (a summation of the parent and fragment BP ions) is positively correlated with the desorption efficiency. A plot of the ion intensity of desorbed BP against the pore size of the chip is shown in Figure 3A (red). Compared with bare ITO slide with the same matrix formula, the chip_5%/10%/30%_ with mesoporous silica membrane as the substrate all exhibited a higher ion desorption efficiency under the same laser intensity, in which the chip_5%_ with the largest pore size afforded the best ion desorption efficiency. To rationalize the effect of different pore sizes on the ion desorption efficiency, the extent of internal energy transfers to BP ions generated by LDI was determined from the SY of the parent BP ions. A plot of the SY against the pore size is illustrated in Figure 3A (blue), in which the SY of parent BP ions decreased with the increase of the silica substrate pore size. Of note, the lower SY equals higher internal energy transfer. Hence, the desorption efficiency (chip_5%_ > chip_10%_ > chip_30%_ > ITO) in general exhibits a consistent trend to the extent of internal energy transfer (chip_5%_ > chip_10%_ > chip_30%_ > ITO), suggesting that increasing the extent of internal energy transfer in the LDI process enhanced the ion desorption efficiency. This phenomenon can be explained by an enhanced thermal desorption mechanism in the chip_5%_, indicating that the increase of the pore size of the silica substrate is positively associated with thermal-driven desorption.

It is reported that the initiator owns a significant effect on the non-thermal driven desorption, as it can perform a phase-transition when heated by the laser, facilitating the desorption of the intact analytes (Northen et al., 2007; Palermo et al., 2018). Therefore, the non-thermal driven desorption would produce more parent BP ions rather than the fragment ion (Tang et al., 2009; Jiang et al., 2021). As shown in Supplementary Figure S17, the survival yield of parent BP is much higher in the IGMSM chip compared to the GMSM chip without the initiator, indicative of the enhanced non-thermal driven desorption through the addition of the initiator. More interestingly, the ion intensity of parent BP desorbed from the different silica substrates with the same amount of gold and initiator are in the order of chip_5%_ > chip_10%_ > chip_30%_ > ITO, suggesting that an enhanced non-thermally driven desorption also existed when the pore size increased (Figure 3B). We then proposed a possible enhanced desorption mechanism for the IGMSM chip: 1) in rapid laser-induced heating of the IGMSM chip, the mesoporous silica substrate with high thermal insulation well reserved the energy, causing a higher chamber temperature in the substrate layer; 2) the trapped heat energy not only bring in a better internal energy transfer for the enhanced thermal desorption but also generated more violent phase-transition of the gold (Supplementary Figure S18) and initiator for enhanced non-thermal desorption (Figure 3B; Figure 3C).

To verify the feasibility in real-case bio-fluids, we first tested the salt tolerance and protein endurance of the optimized IGMSM chip for low-abundance metabolite detection. A mixture containing four small metabolites (proline, lysine, mannitol, and sucrose, 1 mg/mL each), salts (0.5 M NaCl), and proteins (5 mg/mL bovine serum albumin (BSA)) were used to simulate the real sample environment. The characteristic peaks of proline ([M + Na]^+^ [M + 2Na - H]^+^), lysine ([M + Na]^+^ [M + 2Na - H]^+^), mannitol ([M + Na]^+^), and sucrose ([M + Na]^+^) can be detected on the optimized IGMSM chip, highlighting its selectivity and sensitivity for analysis of small metabolites in complex biofluids (Figure 3D). We then achieved the direct metabolic fingerprinting of 1 μL of urine without any pre-treatments. We collected 50 independent metabolic patterns for one KS patient’s urine sample and plotted the heat map, showing that the metabolite signals were distributed vertically and uniformly in a given *m/z* range (Figure 3E). Notably, the features of *m/z* 135.93 and *m/z* 151.91 were normally distributed (*p* > 0.05, Supplementary Figure S19) at a 5% significance level for one urine sample with (Liu et al., 2017) independent patterns respectively, validating the reproducibility of the metabolic pattern extraction (Huang et al., 2020). The above results indicated the reliability and potency of the urine metabolic patterns obtained with IGMSM chip-assisted LDI MS for diagnostic applications.

### 3.4 On-chip *in vitro* metabolic diagnosis of kidney stone patients

**FIGURE 4:**
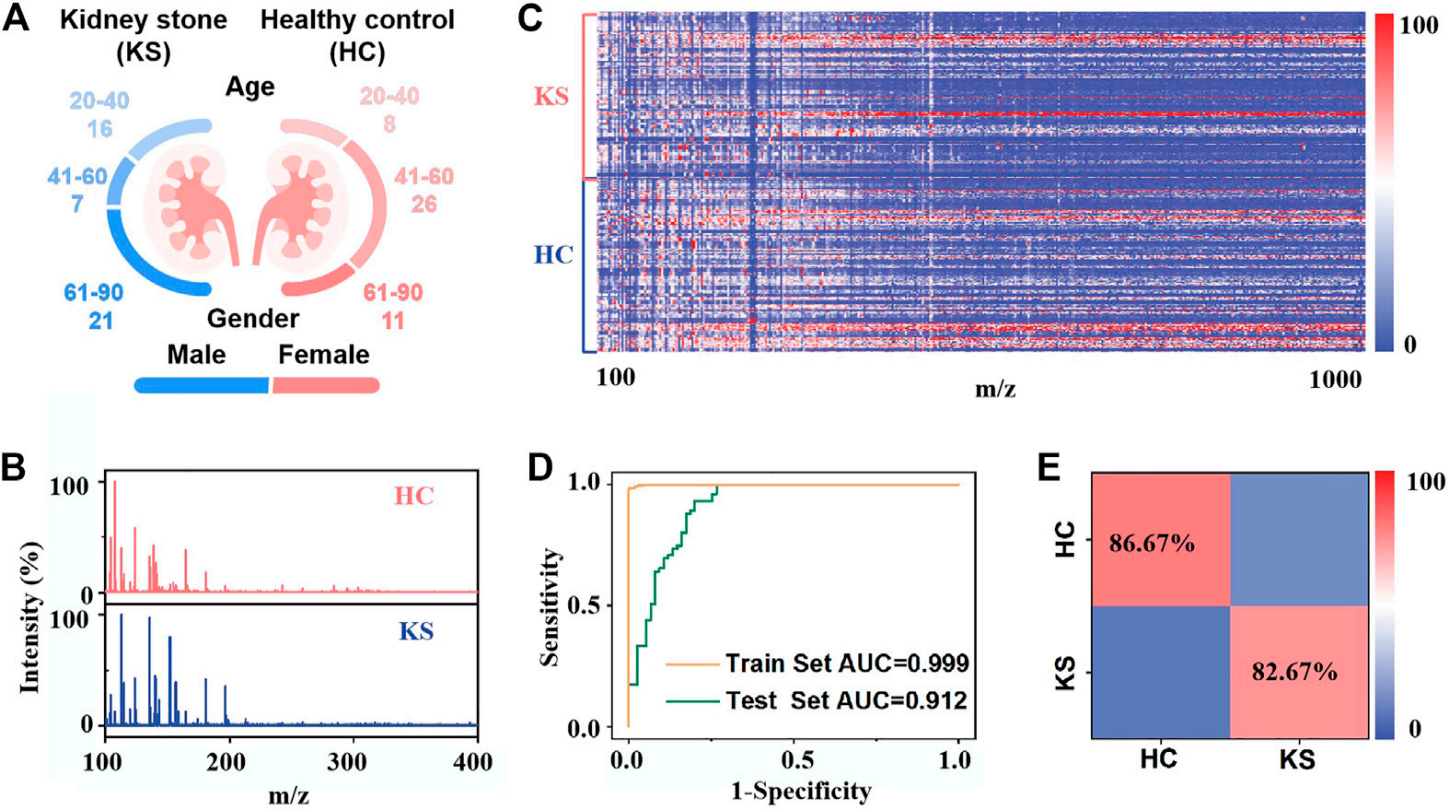
On-chip *in vitro* metabolic diagnosis of KS patients: **(A)** Age distribution of 44 kidney stone patients and 45 healthy controls. **(B)** Typical mass spectra of kidney stone patients and healthy controls. **(C)** Heat map of independent metabolic fingerprinting for urine from 44 kidney stone patients and 45 healthy controls was plotted, using signals within *m/z* range from 100 to 1,000 through data preprocessing. The color scale was processed by logarithmic correction. **(D)** Receiver operating characteristic (ROC) curves based on urine metabolic changes to distinguish KS patients from healthy controls, with an AUC value of 0.999 for the train test (yellow line) and 0.912 for the blind test (green line). **(E)** The confusion matrix from blind test to differentiate 15 KS patients from 15 healthy controls.

We differentiated 44 KS patients from 45 HCs by on-chip metabolic analysis of urine (Figure 4A; Supplementary Table S1), and there was no significant difference in age distribution between the two cohorts (*p* = 0.3654). The typical mass spectra of KS patients and HCs are shown in Figure 4B, in which multiple peaks with distinct differences can be observed in both cases. We recorded the metabolic *m/z* signals of KS patients and HCs in the low mass range (*m/z* of 100–1,000) from urine by the IGMSM chip. We extracted urine metabolic fingerprints (UMFs) from the above 89 KS patients and HCs by preprocessing the original mass spectra. Specifically, the 89 UMFs within *m/z* range from 100–1,000 signals were further organized as the blueprint in Figure 4C, serving as the database for building a diagnostic model. To better elucidate these data, OPLS-DA was applied and displayed the clear group separation based on the UMFs (Supplementary Figure S20, R^2^Y (cum) = 0.883, Q^2^ (cum) = 0.816, *p* < 0.005). Then, we built a diagnostic model and obtained an AUC value of 0.997 with a sensitivity/specificity of 0.9822/1.0000 for the train cohort. (Figure 4D). To elucidate the diagnostic performance of the established model, we conducted blind tests of 30 subjects (KS/HC, 15/15, Supplementary Table S1) with an AUC value of 0.912 with a sensitivity/specificity of 0.8667/0.8267 (Figure 4D). The classification results were quantitatively summarized in a confusion matrix (Figure 4E), and an accuracy of 82.67% was obtained for classifying KS patients *versus* HCs. Permutation tests with 200 iterations were performed for validating the supervised model to avoid overfitting (Supplementary Figure S20). Therefore, the established KS diagnostic model by urine metabolic fingerprint can successfully differentiate KS patients and HCs, proving the established on-chip *in vitro* metabolic screening enjoys high throughput and considerable accuracy.

KS is a common disease in urology and lays a heavy economic burden on patients worldwide (Lieske et al., 2006; Rule et al., 2020; Abufaraj et al., 2021). The diagnosis of the KS is mainly based on ultrasonography (Pearle Margaret et al., 2014; Bultitude et al., 2016), and the etiology of the disease remains unclear (Alelign and Petros, 2018; Howles and Thakker, 2020; Halbritter, 2021). Thus, how to prevent stone formation and recurrence, as well as predicting its potential risk, remains a big challenge (Hyams and Matlaga, 2015). Urine tests promise easy and accurate diagnosis of KS for the simple measurement using urine samples and low costs for point-of-care testing, which is non-invasive and facile for universal applications (Yang et al., 2020; Ding et al., 2022). Furthermore, the biological predictors from urine can better interpret the underlying molecular mechanisms for etiology (Maiuolo et al., 2016; Noone et al., 2018; Ståhl et al., 2019). Considering the metabolic disturbances in KS as demonstrated by previous reports (Khan et al., 2016; Sorokin et al., 2017), our results offer new insights for *in vitro* diagnosis of KS by direct metabolic fingerprinting of urine. Though many studies also attempted to construct the MS-based platform for the KS differentiation (Liu et al., 2012; Chao et al., 2018; Wang et al., 2019), our established on-chip LDI MS platform is highlighted due to the minimum sample pre-treatment and the convenience for large-scale screening. In traditional MS-based metabolomics, tedious sample pre-treatments (at least 0.5–1 h for each sample) are indispensable to address the low abundance of metabolites and interference of salts and proteins in bio-samples. These time-consuming procedures may cause sample information loss and high overhead, hindering the universal application of conventional MS techniques in clinics. By comparison, our approach directly profiled urine metabolites (∼1 μL) with the enhanced LDI efficacy in seconds, exhibiting high efficiency and feasibility for real clinic use.

## 4 Conclusion

In conclusion, we constructed a ternary component MS chip for metabolic fingerprinting-based IVD, which can considerably improve the desorption efficiency through thermally and non-thermally driven desorption in LDI MS detection. The chip performance was optimized by tuning the pore size of silica membranes for better thermal insulation, the sputtering time of gold for specific nanogaps and surface plasmon resonance, and the initiator incorporating amount for suisable surface wettability. The urine metabolic fingerprints directly extracted from one IGMSM chip allowed the discrimination of KS patients from the controlled subjects. Our work makes solid contributions to designing nano-material-based platforms for advanced metabolic analysis toward precision medicine.

## Data Availability

The original contributions presented in the study are included in the article/Supplementary Material, further inquiries can be directed to the corresponding author.
