# Supplementary material for: Construction of a ternary component chip with enhanced desorption efficiency for laser desorption/ionization mass spectrometry based metabolic fingerprinting
Source: Front Bioeng Biotechnol. 2023 Jan 20;11:1118911. doi: 10.3389/fbioe.2023.1118911 (PMC9895787; doi:10.3389/fbioe.2023.1118911)
Supplement: Supplementary file 1 [file DataSheet1.docx]

Supplementary Material

Construction of a ternary component chip with enhanced desorption efficiency for laser desorption/ionization mass spectrometry based metabolic fingerprinting

Yajie Ding^1+^, Congcong Pei^1+^, Kai Li^2+^, Weikang Shu^1^, Wenli Hu^1^, Rongxin Li^1^, Yu Zeng^1^ and Jingjing Wan^1^*

*** Correspondence:** Jingjing Wan: jjwan@chem.ecnu.edu.cn

# Supplementary Figures and Tables

## Supplementary Figures

**
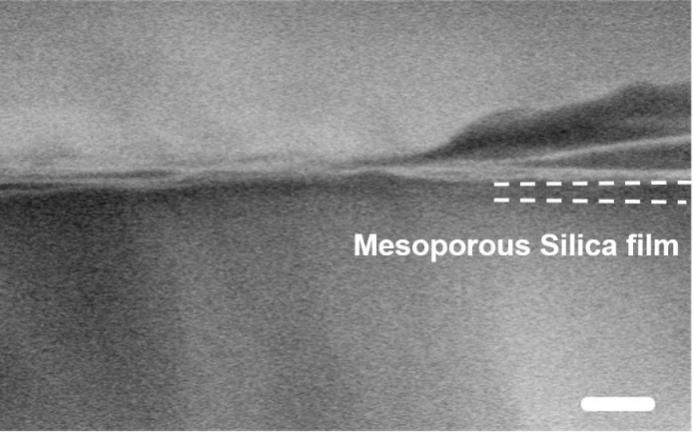
**

**Supplementary Figure S1.** Section image of SEM of mesoporous silica membrane on ITO chip (Scale bar is 100 nm).

**
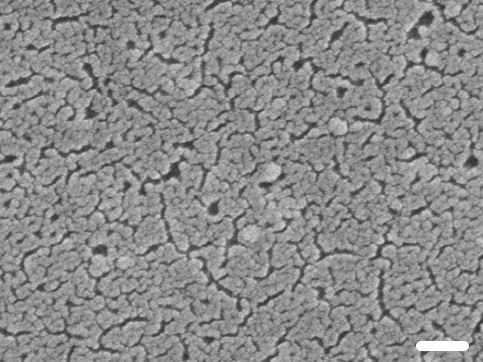
**

**Supplementary Figure S2**. Top-view image of SEM of IGMSM chip (scale bar is 100 nm).

**
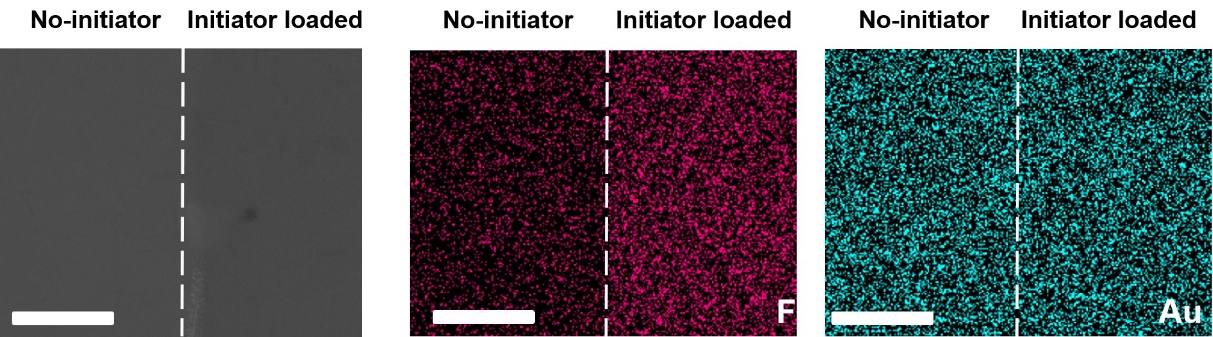
**

**Supplementary Figure S3**. Section mapping images of regions with or without initiators on the chip (scale bar is 100 μm).

**
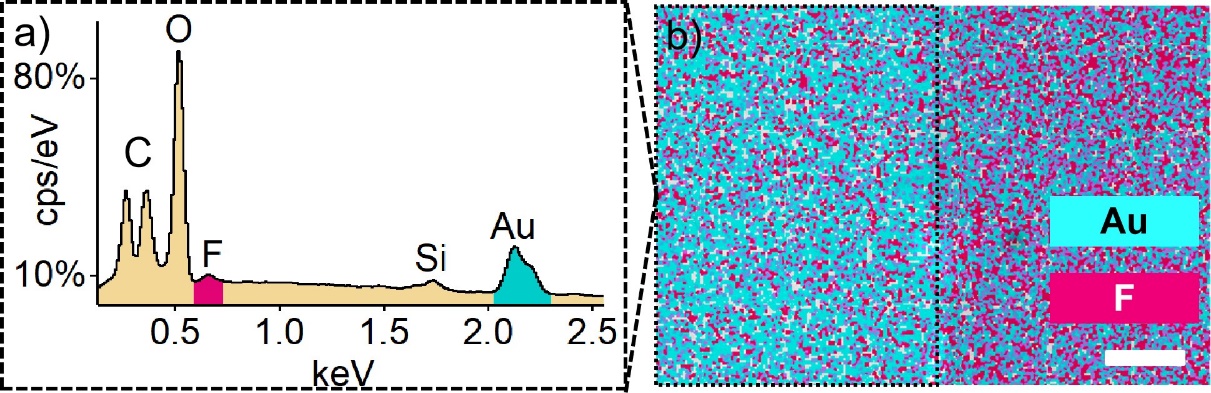
**

**Supplementary Figure S4**. a) EDS spectra of the area selected in right figure. b) Corresponding element mapping images of the IGMSM chip (scale bar is 100 μm).


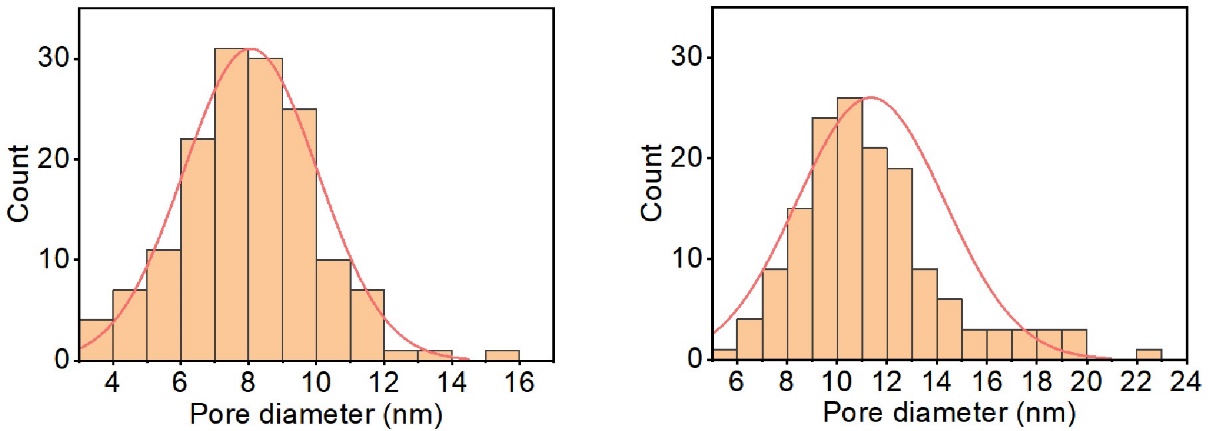


**Supplementary Figure S5**. Pore diameter distribution of silica membranes of chip_10%_ and chip_5%_.


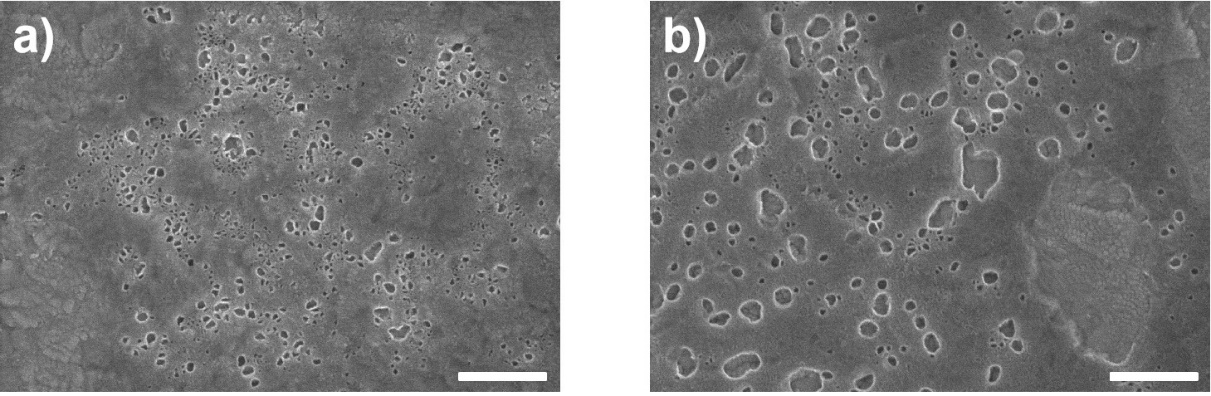


**Supplementary Figure S6**. Top-view image of SEM of mesoporous silica membranes on ITO chips using a) 3% (v/v) and b) 1% (v/v) TEOS / cyclohexane (Scale bar is 200 nm).

**
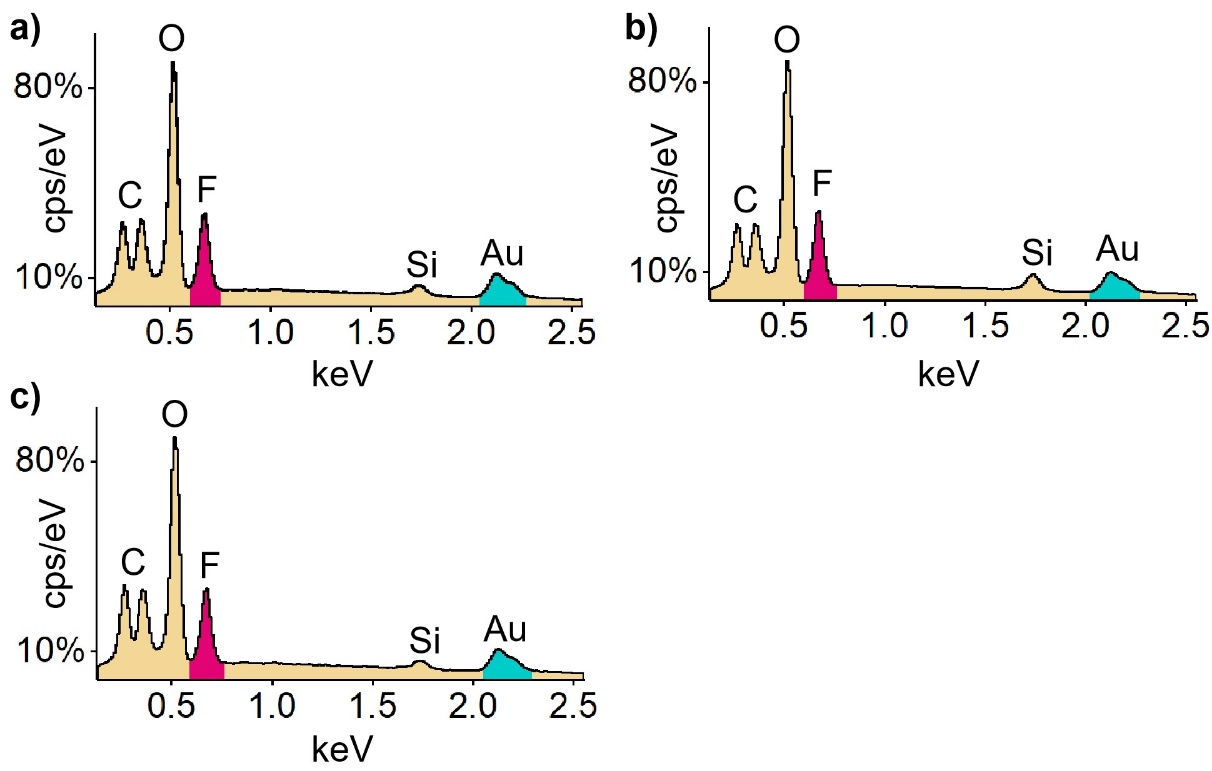
**

**Supplementary Figure S7**. EDS spectra of a) chip_10%_, b) chip_30%_, and c) chip made by bare ITO.


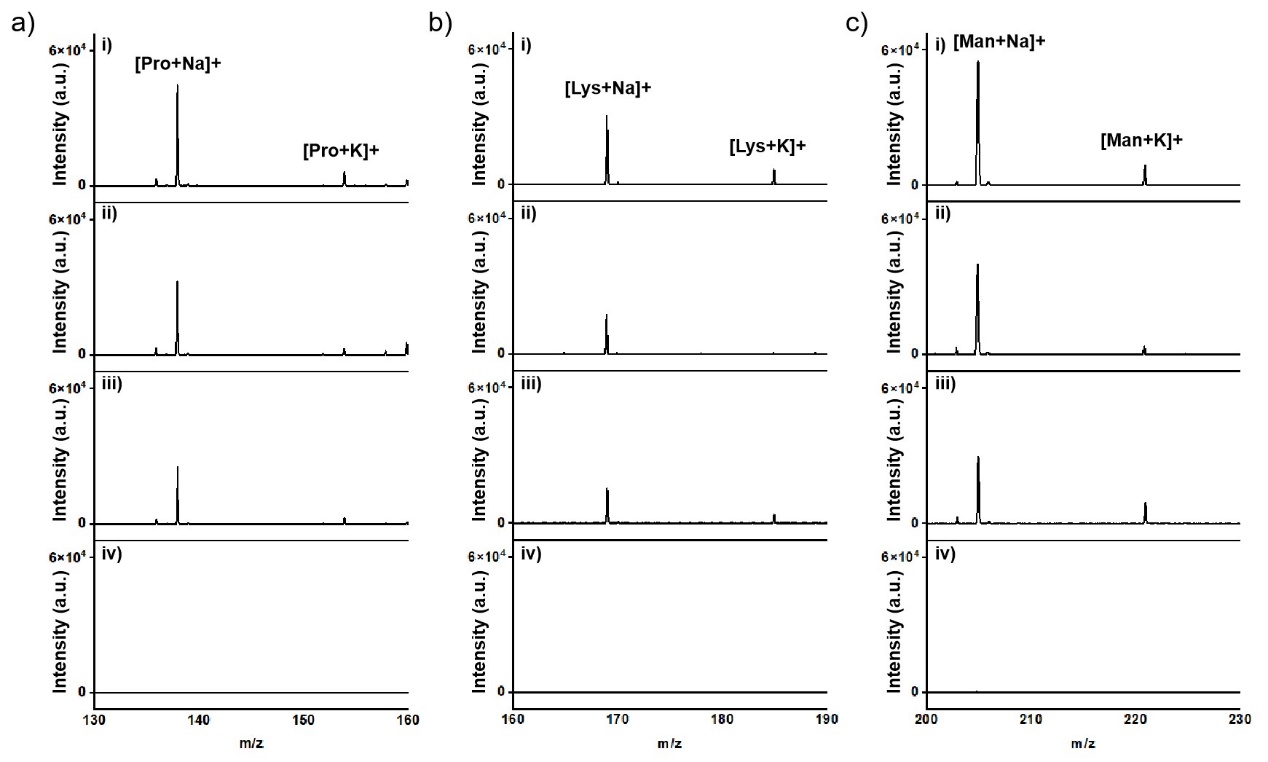


**Supplementary Figure S8**. Typical mass spectra of 1 mg/mL a) proline, b) lysine, and c) mannitol with on i) chip_5%_, ii) chip_10%_, iii) chip_30%_, and iv) chip made by bare ITO.


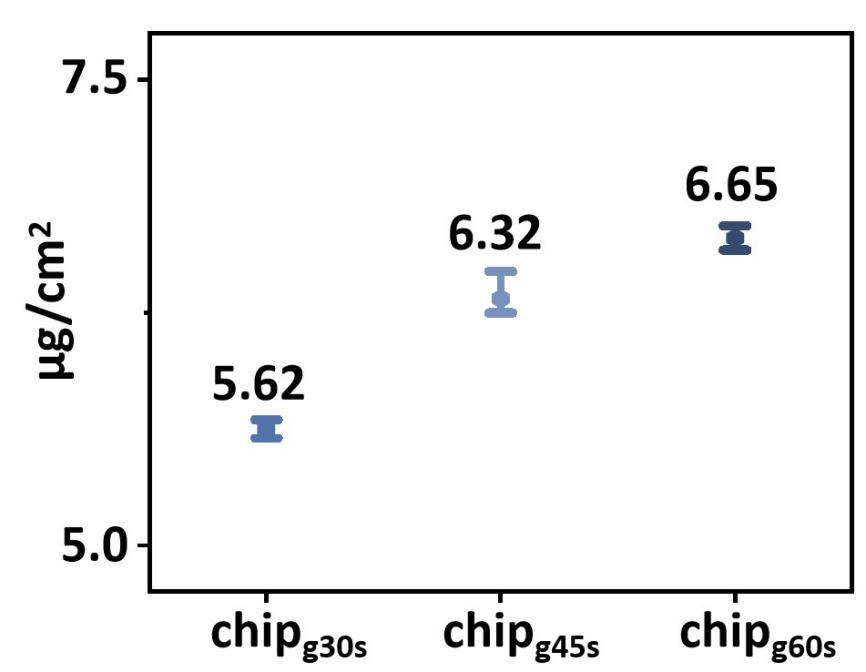


**Supplementary Figure S9**. Surface Au density of chip_g30s_, chip_g45s_, and chip_g60s_.


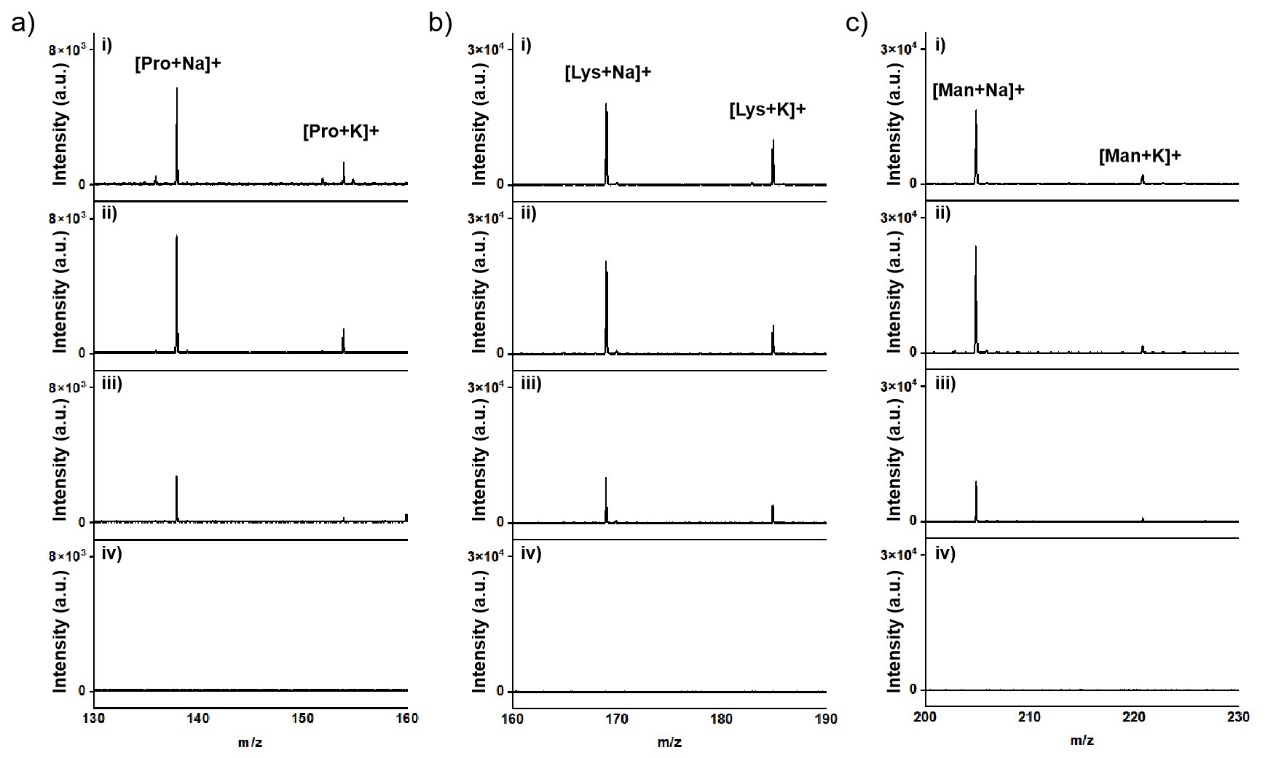


**Supplementary Figure S10**. Typical mass spectra of 1 mg/mL a) proline, b) lysine, and c) mannitol with on i) chip_g60s_, ii) chip_g45s_, iii) chip_g30s_, and iv) chip without Au layer.


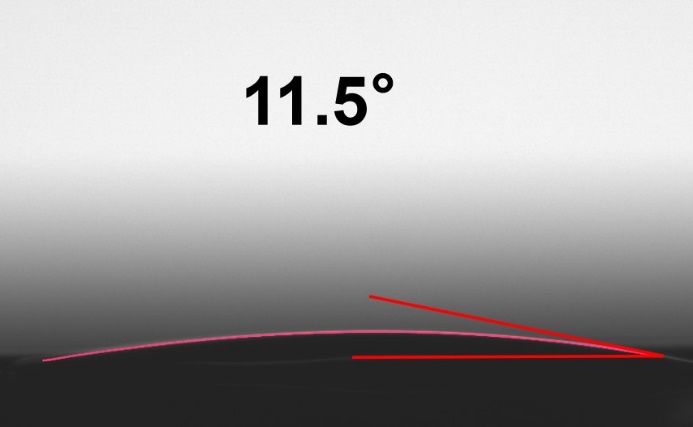


**Supplementary Figure S11**. The contact angle of the chip without initiator.


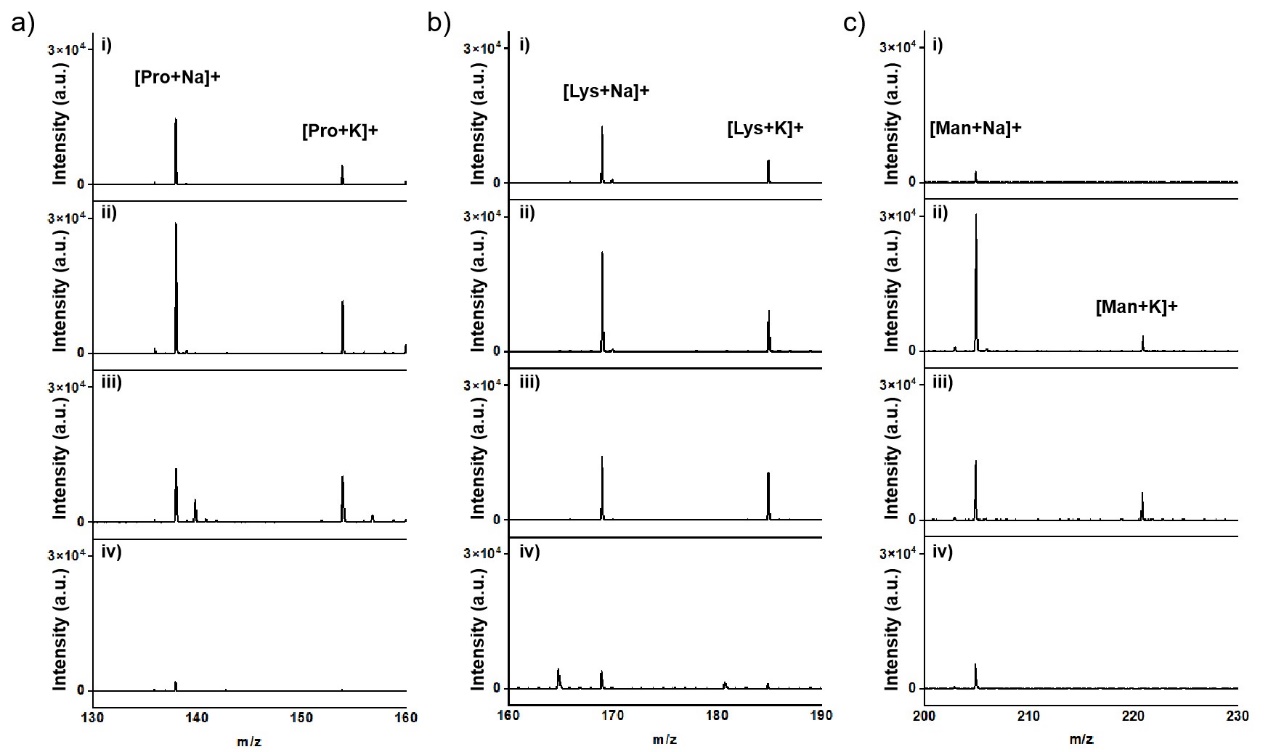


**Supplementary Figure S12**. Typical mass spectra of 1 mg/mL a) proline, b) lysine, and c) mannitol with on i) chip_i90s_, ii) chip_i60s_, iii) chip_i30s_, and iv) chip without initiator.


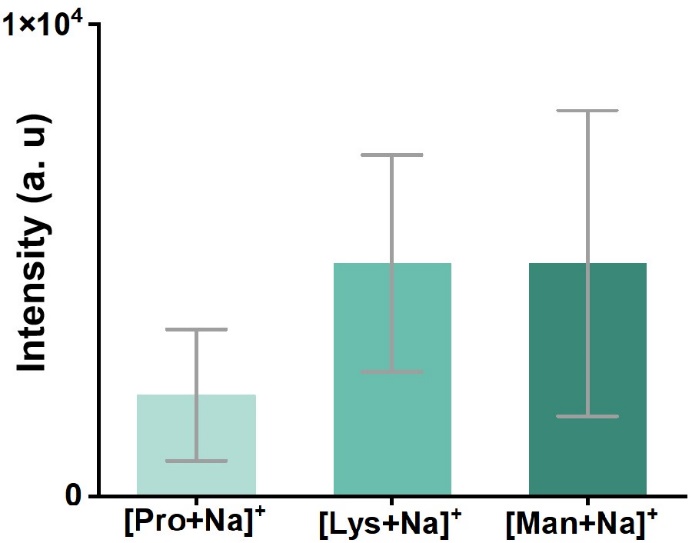


**Supplementary Figure S13**. Mean intensities of Na adducted peaks for 1 mg/mL proline, lysine, and mannitol on the chip without initiator.


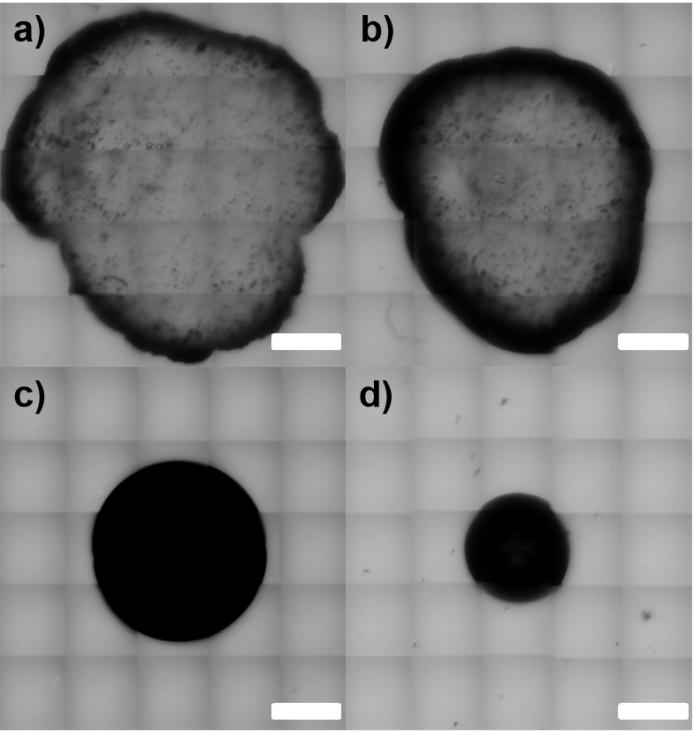


**Supplementary Figure S14**. Microscope images of 1 μL 1 mg/mL fluorescein sodium spots on the a) chip without initiator, b) chip_i30s_, c) chip_i60s_, and d) chip_i90s_ (Scale bar is 600 um).


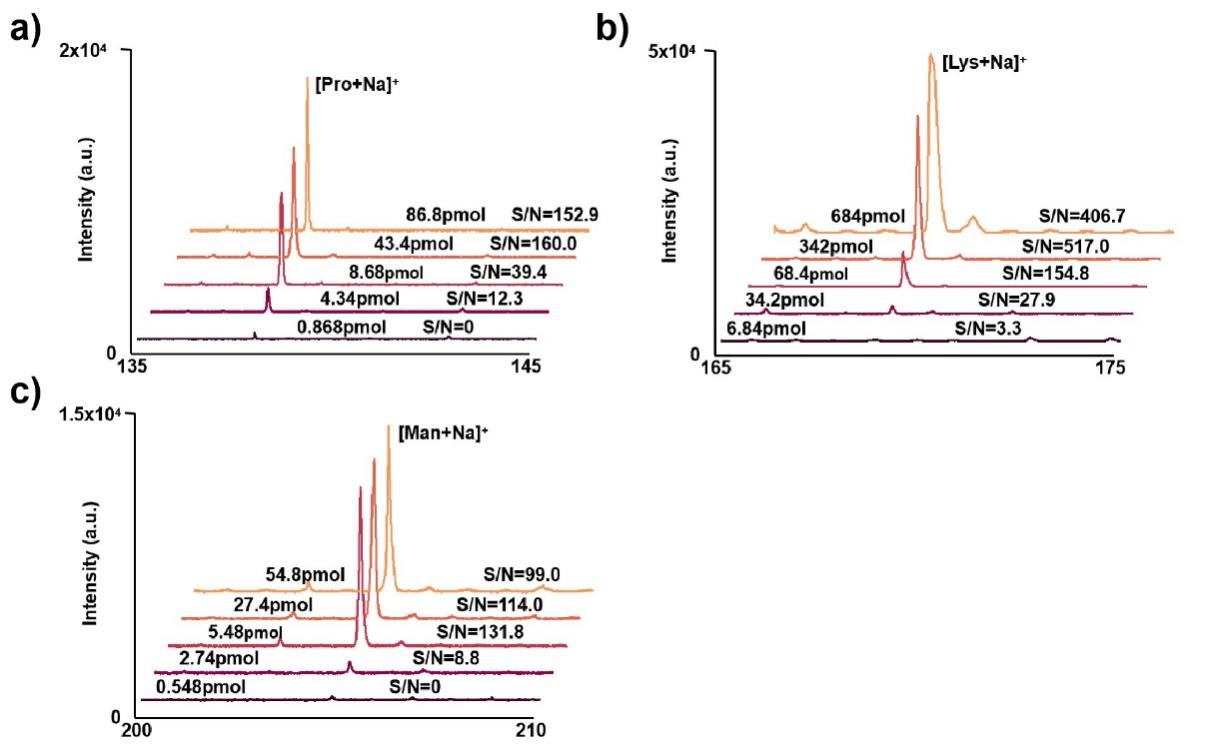


**Supplementary Figure S15**. Typical mass spectra of a) proline, b) lysine and c) mannitol at a different amount by IGMS chip.


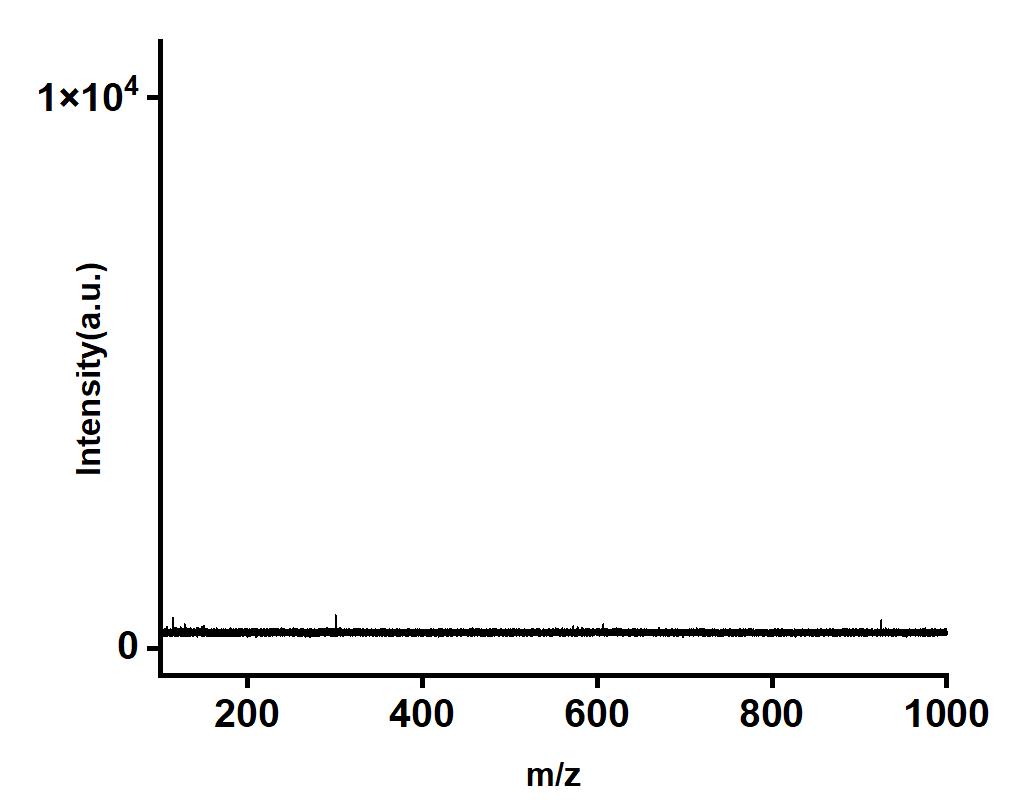


**Supplementary Figure S16.** The background signal of the bare IGMSM chip.


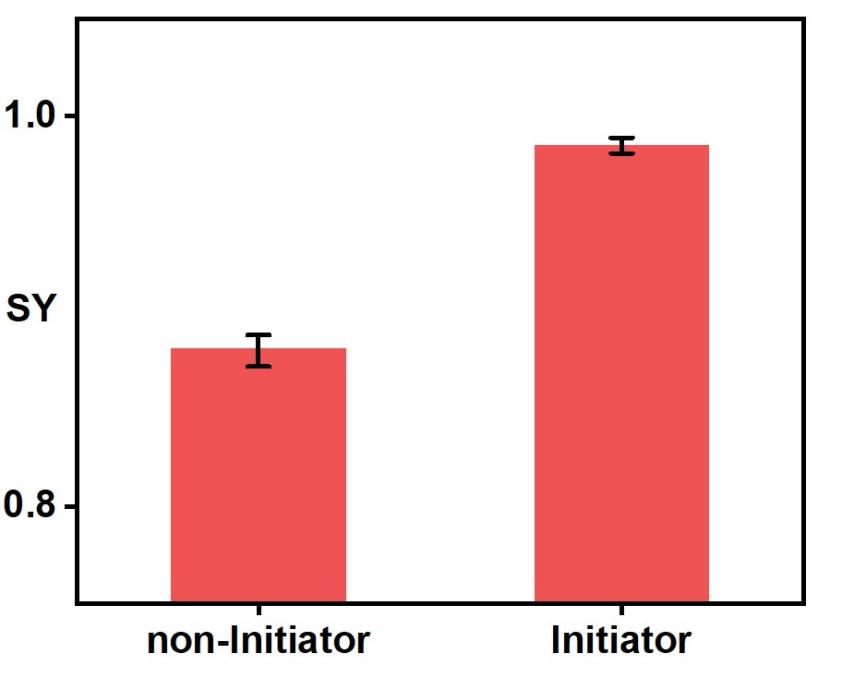


**Supplementary Figure S17**. The SY of parent ions desorbed from chips with or without initiators.


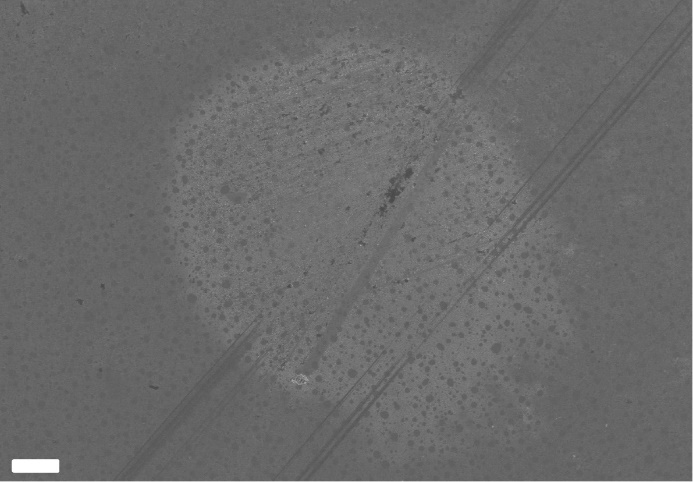


**Supplementary Figure S18**. Top-view images of SEM of a spot on the IGMSM chip after laser irradiation (scale bar is 10 μm).


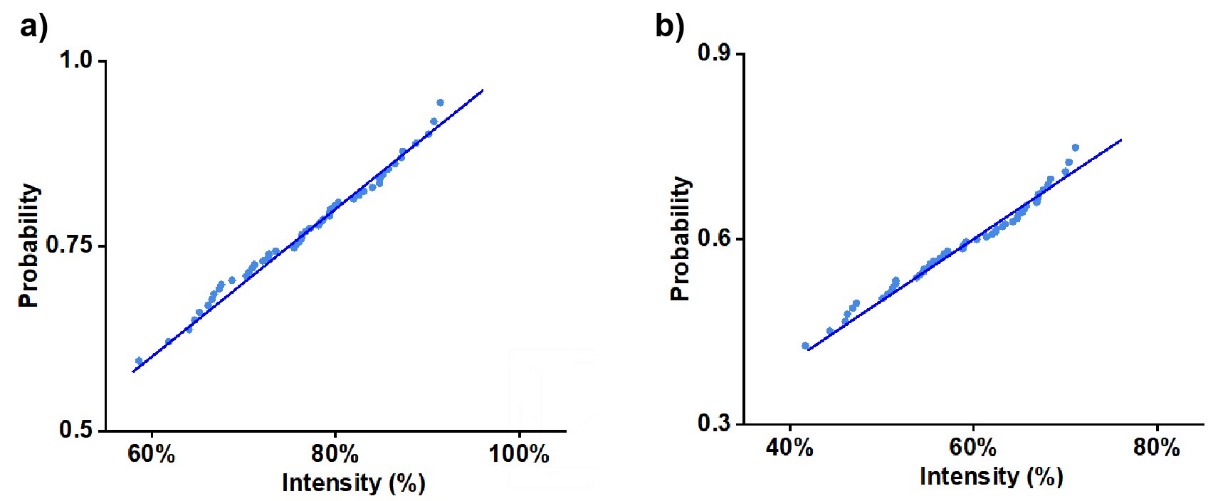


**Supplementary Figure S19**. Probability of a normal distribution of *m/z* features at a) 135.93 and b) 151.91 for 50 patterns of one urine sample from KS patients, both with p > 0.05 (n = 50 independent experiments, two-sided Lilliefors (Kolmogorov–Smirnov) test with no adjustment made for multiple comparisons). Lines are the reference lines for normal distribution.


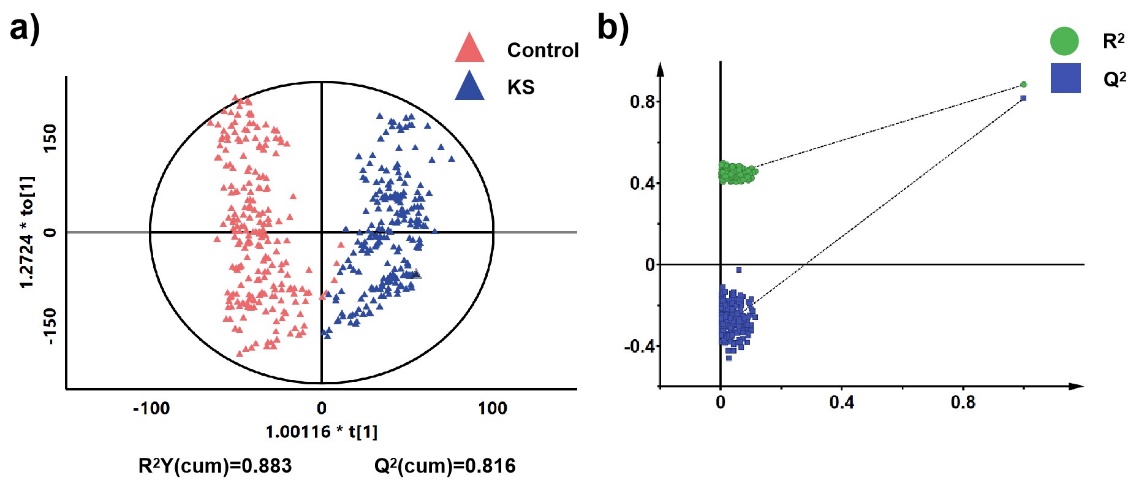


**Supplementary Figure S20**. a) OPLS-DA score plots showing the global metabolic difference between KS patients and HC (p < 0.005); b) The Permutations Plot for OPLS-DA of KS patients vs HC.

## Supplementary Tables

|  | chip_5%_ | chip_10%_ | chip_30%_ | chip made by bare ITO |
| --- | --- | --- | --- | --- |
| Au (wt%) | 55.1% | 54.2% | 57.4% | 54.0% |
| F (wt%) | 15.2% | 14.3% | 17.7% | 14.9% |

**Supplementary Table S1.** Contents of Au and F on the chip_5%_, chip_10%_, chip_30%_, and chip made by bare ITO by using indium from ITO glass as the internal standard.

| Matrices/Chips | Instrument model | Analytes | Reproducibility  (RSD%) | Sensitivity  (LOD) | Citation |
| --- | --- | --- | --- | --- | --- |
| IGMSM Chip | Autoflex MALDI-TOF/TOF (Bruker) | proline, lysine and mannitol | 4.3-8.7% | 2-34.2 pmol | This paper |
| PdAu@Au concave nanocubes | Autoflex MALDI-TOF/TOF (Bruker) | alanine, glucose, lysine, mannitol, and phenylalanine | 3.28% | 10-50 pmol | (Shu et al.) |
| Interfacial self-assembled 2D NP arrays | MALDI-TOF/TOF (AB SCIEX 5800) | glucose | 2.9% | 1000 pmol | (Wang et al., 2021) |
| gold@graphitized mesoporous silica nanocomposite | MALDI-TOF/TOF (AB SCIEX 5800) | phenylalanine, arginine, tryptophan, and histidine | 5.5% | 5-10 pmol | (Xu et al., 2015) |
| CuFe_2_O_4_ magnetic nanocrystal clusters | Bruker Autoflex II mass spectrometer (Bruker) | phenylalanine | 12.1% | 5 pmol | (Lin et al., 2015) |
| Spherical COF-V | Bruker Autoflex II mass spectrometer (Bruker) | glucose | 8.79% | 1 pmol | (Ouyang et al., 2020) |

**Supplementary Table S2.** Reproducibility and sensitivity of different matrices/chips.

Lin, Z., Zheng, J., Bian, W., and Cai, Z. (2015). CuFe2O4 magnetic nanocrystal clusters as a matrix for the analysis of small molecules by negative-ion matrix-assisted laser desorption/ionization time-of-flight mass spectrometry. *Analyst* 140(15), 5287-5294. doi: 10.1039/C5AN00625B.

Ouyang, D., Luo, K., Ma, W., Wu, J., Li, J., He, Y., et al. (2020). A spherical covalent-organic framework for enhancing laser desorption/ionization mass spectrometry for small molecule detection. *Analyst* 145(8), 3125-3130. doi: 10.1039/D0AN00171F.

Shu, W., Zhang, M., Zhang, C., Li, R., Pei, C., Zeng, Y., et al. An Alloy Platform of Dual-Fingerprints for High-Performance Stroke Screening. *Adv. Funct. Mater.*, 2210267. doi: https://doi.org/10.1002/adfm.202210267.

Wang, Y., Zhang, K., Tian, T., Shan, W., Qiao, L., and Liu, B. (2021). Self-Assembled Au Nanoparticle Arrays for Precise Metabolic Assay of Cerebrospinal Fluid. *ACS Appl. Mater. Interfaces* 13(4), 4886-4893. doi: 10.1021/acsami.0c20944.

Xu, G., Liu, S., Peng, J., Lv, W., and Wu, R.a. (2015). Facile Synthesis of Gold@Graphitized Mesoporous Silica Nanocomposite and Its Surface-Assisted Laser Desorption/Ionization for Time-of-Flight Mass Spectroscopy. *ACS Appl. Mater. Interfaces* 7(3), 2032-2038. doi: 10.1021/am507894y.

**Supplementary Table S3.** Summary of 45 HC and 44 KS patients for urine fingerprinting for train test.

| **Subjects** | **Sex** | **Age** | **Treated** | **Subjects** | **Sex** | **Age** | **Treated** |
| --- | --- | --- | --- | --- | --- | --- | --- |
| KS patient | Male | 31 | NA | Control | Male | 58 | NA |
| KS patient | Male | 73 | NA | Control | Male | 67 | NA |
| KS patient | Male | 51 | NA | Control | Male | 67 | NA |
| KS patient | Male | 56 | NA | Control | Male | 63 | NA |
| KS patient | Male | 39 | NA | Control | Male | 21 | NA |
| KS patient | Male | 29 | NA | Control | Male | 34 | NA |
| KS patient | Male | 48 | NA | Control | Male | 47 | NA |
| KS patient | Male | 51 | NA | Control | Male | 31 | NA |
| KS patient | Male | 54 | NA | Control | Male | 59 | NA |
| KS patient | Male | 52 | NA | Control | Male | 62 | NA |
| KS patient | Male | 44 | NA | Control | Male | 36 | NA |
| KS patient | Male | 63 | NA | Control | Male | 32 | NA |
| KS patient | Male | 63 | NA | Control | Male | 65 | NA |
| KS patient | Male | 34 | NA | Control | Male | 61 | NA |
| KS patient | Male | 57 | NA | Control | Male | 40 | NA |
| KS patient | Male | 47 | NA | Control | Male | 58 | NA |
| KS patient | Male | 54 | NA | Control | Male | 43 | NA |
| KS patient | Male | 60 | NA | Control | Male | 33 | NA |
| KS patient | Male | 27 | NA | Control | Male | 61 | NA |
| KS patient | Male | 52 | NA | Control | Male | 28 | NA |
| KS patient | Male | 59 | NA | Control | Male | 62 | NA |
| KS patient | Male | 55 | NA | Control | Male | 64 | NA |
| KS patient | Male | 46 | NA | Control | Male | 64 | NA |
| KS patient | Male | 58 | NA | Control | Male | 63 | NA |
| KS patient | Male | 55 | NA | Control | Male | 21 | NA |
| KS patient | Male | 52 | NA | Control | Male | 65 | NA |
| KS patient | Male | 46 | NA | Control | Male | 64 | NA |
| KS patient | Male | 60 | NA | Control | Male | 64 | NA |
| KS patient | Female | 54 | NA | Control | Male | 61 | NA |
| KS patient | Female | 68 | NA | Control | Female | 87 | NA |
| KS patient | Female | 69 | NA | Control | Female | 62 | NA |
| KS patient | Female | 52 | NA | Control | Female | 20 | NA |
| KS patient | Female | 48 | NA | Control | Female | 66 | NA |
| KS patient | Female | 54 | NA | Control | Female | 22 | NA |
| KS patient | Female | 34 | NA | Control | Female | 65 | NA |
| KS patient | Female | 64 | NA | Control | Female | 35 | NA |
| KS patient | Female | 54 | NA | Control | Female | 20 | NA |
| KS patient | Female | 74 | NA | Control | Female | 65 | NA |
| KS patient | Female | 37 | NA | Control | Female | 21 | NA |
| KS patient | Female | 76 | NA | Control | Female | 50 | NA |
| KS patient | Female | 69 | NA | Control | Female | 66 | NA |
| KS patient | Female | 58 | NA | Control | Female | 40 | NA |
| KS patient | Female | 75 | NA | Control | Female | 58 | NA |
| KS patient | Female | 33 | NA | Control | Female | 62 | NA |
|  |  |  |  | Control | Female | 21 | NA |

**Supplementary Table S4.** Summary of 15 HC and 15 KS patients for urine fingerprinting for blind test.

| **Subjects** | **Sex** | **Age** | **Treated** | **Subjects** | **Sex** | **Age** | **Treated** |
| --- | --- | --- | --- | --- | --- | --- | --- |
| KS patient | Male | 75 | NA | Control | Male | 52 | NA |
| KS patient | Male | 74 | NA | Control | Male | 63 | NA |
| KS patient | Male | 36 | NA | Control | Male | 62 | NA |
| KS patient | Male | 58 | NA | Control | Male | 66 | NA |
| KS patient | Male | 57 | NA | Control | Male | 65 | NA |
| KS patient | Male | 27 | NA | Control | Male | 35 | NA |
| KS patient | Male | 53 | NA | Control | Male | 57 | NA |
| KS patient | Male | 69 | NA | Control | Male | 65 | NA |
| KS patient | Male | 60 | NA | Control | Male | 55 | NA |
| KS patient | Female | 69 | NA | Control | Female | 32 | NA |
| KS patient | Female | 69 | NA | Control | Female | 65 | NA |
| KS patient | Female | 55 | NA | Control | Female | 32 | NA |
| KS patient | Female | 58 | NA | Control | Female | 47 | NA |
| KS patient | Female | 71 | NA | Control | Female | 63 | NA |
| KS patient | Female | 56 | NA | Control | Female | 81 | NA |
